# Toward an Ecology of Household Biomes

**DOI:** 10.1111/ele.70462

**Published:** 2026-10-06

**Authors:** Raul Costa‐Pereira, Robert R. Dunn

**Affiliations:** ^1^ Departamento de Biologia Animal Universidade Estadual de Campinas (Unicamp) São Paulo Brazil; ^2^ Department of Applied Ecology North Carolina State University Raleigh North Carolina USA

**Keywords:** eco‐socio‐evo dynamics, ecosystem services, food webs, individual variation, indoor, urban biodiversity, urban ecology

## Abstract

Understanding the overlooked ecology of our homes can provide ecologists with unprecedented data and fresh ideas while also informing broader societal concerns. By studying the biodiversity we interact with most often, ecologists can foster appreciation for the significance of ecological and evolutionary insights in our everyday lives.

## Graphical abstract

We encourage ecologists to explore the neglected ecology of our homes and how biodiversity and ecological interactions vary among households with diverse habits and characteristics. By studying the biodiversity we interact with most often, ecologists can foster appreciation for the significance of ecological and evolutionary insights in our everyday lives.

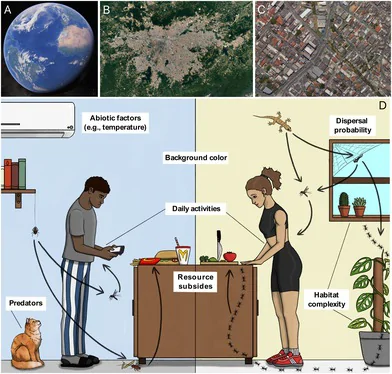

Ecologists made a conscious decision early in the history of the field to focus on regions that were more removed from human influence (Kingsland 2005). As a result, many of the classic examples of ecology and evolution come from ecosystems where the human footprint is, if not absent, lighter. In addition to focusing on remote ecosystems, ecologists have also tended to focus on larger and conspicuous species and, among those species, taxa that are charismatic (given the peculiarities of human senses and aesthetics) (Lorimer 2007; Dapporto et al. 2026). However, nearly all ecological and evolutionary processes that occur in remote locales also occur in those that are more immediate, including indoors. We hypothesise that the focus of ecology and evolution on the remote has had many unintended consequences. It has meant that many of the most well‐understood insights from ecology and evolution are disconnected from human daily life and policymakers' decisions. Here, we advocate that studying the ubiquitous but neglected ecology of our homes can not only bring unprecedented data and new ideas to ecologists but also help connect people with biodiversity and foster an understanding of the value of ecological and evolutionary insights in our daily lives. It also has the potential to engage an underutilised asset, namely the hidden expertise of many people about their own (ecological) experiences (Gaston et al. 2018).

## 1 The Biodiversity We Interact with the Most

Since the early 1900s, it has been clear that humans have and depend upon bodily microbiomes. The web of interactions with species living in homes but off the body has been slower to be noted (Martin et al. 2015), at least by Western science (conversely, poets and other writers have long been concerned with the life of interiors) (Dunn 2018). The ecological realm comprising species that reside and can (although do not necessarily always) reproduce in enclosed and semi‐enclosed built structures is far more diverse than often thought. Although household pests (e.g., flies, mosquitoes, ants, cockroaches) tend to be the most evident components of household biotas, molecular methods have revealed thousands of species of bacteria, fungi and arthropods that live inside houses (Barberán et al. 2015; Madden et al. 2016; Gamez et al. 2026). A comprehensive year‐round survey documented over 1000 macroscopic animal species, both indoors and outdoors, in a single home in Australia (Rogers et al. 2024). Some of these species feed on our food, while others feed or scavenge on the species that consume our food (Figure 1) or use our homes as a source of shelter and favourable environmental conditions (Bertone et al. 2016; Leong et al. 2017; Allf et al. 2026). However, current evidence on the diversity of indoor organisms comes from a handful of studies in the global North (but see Rogers et al. 2024), and surprisingly, we still know very little about how many and which species coexist with humans in homes around the world. We know even less about how these species interact with one another and with us. Even as we appreciate that the presence of some indoor species brings benefits to human well‐being, the absence of others reduces well‐being, and the presence of yet others reduces well‐being.

**FIGURE 1:**
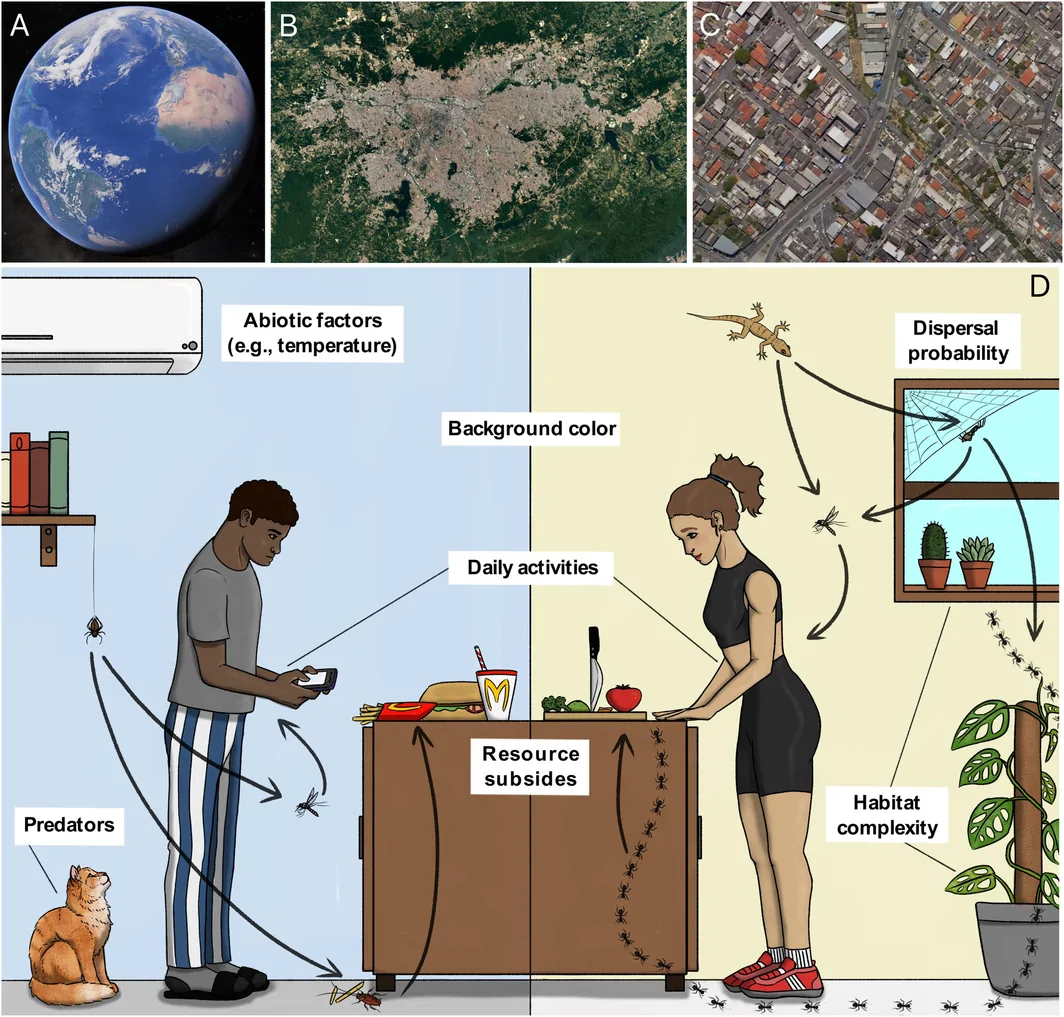
Ecologists have traditionally sought to uncover biodiversity patterns across large biogeographic scales (A), primarily focusing on areas with minimal human impact. Recently, urban ecologists have added nuance to the traditional focus on how urbanization affects wildlife (B) by investigating how biodiversity varies within complex urban mosaics (C). Here, we encourage ecologists to collaborate to explore the ubiquitous yet neglected ecology of our homes and how biodiversity and ecological interactions vary among households with diverse profiles, habits, and characteristics (D). Satellite images (A–C) are from Google Earth (2025 Google). Panels (B) and (C) depict the megacity of São Paulo, Brazil. Panel (D) was created by Felipe C. Coelho.

## 2 Houses as Replicated Ecological Experiments

Although built space in cities is often viewed as a homogeneous grey landscape for biodiversity, it comprises a complex environmental mosaic shaped by ecological, social, and environmental forces (Des Roches et al. 2020; Schell et al. 2020; Carlen et al. 2025). Part of this heterogeneity stems from the spatial organization of cities and other settlements, which creates environmental differences across neighbourhoods. While ecologists have traditionally focused on the effects of physical factors (e.g., green areas, streetlights, impervious surfaces) on urban biodiversity patterns, social and political factors have recently garnered significant attention (Schell et al. 2020; Poulton Kamakura et al. 2024; Carlen et al. 2025). However, environmental variation relevant for indoor biodiversity can also emerge in a much finer‐grained form within cities.

From an ecological perspective, houses can be seen as habitat patches for indoor biodiversity bounded by walls. Compared to the outdoors, houses have more predictable conditions (e.g., temperature, humidity) (Martin et al. 2015) and concentrate highly energetic and nutritious resources (e.g., human and pet food). Even within the same neighbourhood, houses can diverge substantially in their environmental context for indoor biodiversity (Figure 1). Therefore, we argue that ecologists can leverage houses as numerous replicates in a global “natural” experiment to understand and predict patterns in the composition and diversity of household species. Indeed, key processes that drive community patterns (Vellend 2010)—for example, selection, drift, and dispersal—can not only be used to investigate how urbanization impacts biodiversity, but also to understand how household biomes vary within and between cities.

Environmental variation between homes is at least partially related to physical attributes of these habitat patches, such as property size, architectural style, interior design, and amenities. For example, in houses with air conditioning, microscopic and macroscopic indoor biodiversity experience thermal conditions different from those in houses without climate control. Additionally, the presence of a basement, backyard, attic, or even houseplants can affect indoor biodiversity by providing habitat heterogeneity (Bertone et al. 2016; Leong et al. 2017; Gamez et al. 2026). Therefore, environmental factors classically noted by ecologists as key drivers of biodiversity variation—such as habitat complexity, environmental stability, and temperature—can vary widely across houses (Figure 1), creating opportunities to test how habitat structure and abiotic factors shape biodiversity patterns even within the same neighbourhood.

Beyond these physical effects, humans exhibit tremendous intraspecific functional variation that can influence the biodiversity of our homes. Households can consist of, for example, strict herbivores to highly carnivorous individuals, diurnal to nocturnal workers, and highly social to solitary people. Human individuals are unique in their habits and characteristics. Many households also include pet species, which has implications for other non‐human indoor species (Barberán et al. 2015; Dannemiller et al. 2016; Madden et al. 2016). Thus, individuals' decisions create fine‐scale heterogeneity across houses that has been largely overlooked as a potential driver of biodiversity patterns and processes, such as community structure and energy flow in urban ecosystems. For instance, ecologists have long been interested in how the latitudinal productivity gradient shapes diversity patterns and food web functioning. On a much smaller scale (e.g., Norbiatto et al. 2026), resource availability to consumers can differ strikingly between neighbours, who, for instance, may have all meals at restaurants or always cook at home. In this sense, from the perspective of indoor biological communities, human actions and habitats, along with their astonishing variation across households, can significantly shape biodiversity patterns. This perspective can offer a mechanistic understanding of urban biota and provide practical, accessible study systems to address enduring questions in ecology.

## 3 Toward a Macroecology of Household Biomes

Although we are still surprisingly in the early stages of understanding the biodiversity of homes, conceptual and applied advances in this area require more than just independent research focused on specific urban settlements. A substantial challenge to achieving a macroecological understanding of household biomes is documenting indoor biodiversity in regions where studies are incipient or nonexistent, particularly in the tropics. Although most people and houses are in tropical regions, few house studies come from low latitudes. Africa and South America together have more than 380 million households, but studies documenting indoor biodiversity are clearly missing. Importantly, the biodiversity, natural history and ecological dynamics of tropical houses are likely to differ from those in the Global North for multiple environmental and cultural reasons. This lack of data from megadiverse biogeographic regions hampers our ability to understand and predict the macroecology of household biodiversity. Even the most basic questions, such as “Are tropical houses megadiverse?” are currently impossible to answer because of the strong geographic biases in available data. Coordinating large‐scale projects across multiple cities and biogeographical regions is essential to reveal global patterns of household biodiversity and their links to human lives across cultures.

## 4 Household Ecosystem Services and Disservices

Beyond the nontrivial challenge of documenting the taxonomic diversity of household biomes, we are still far from fully understanding their direct and indirect effects on human well‐being, both positive and negative. Household biota have historically been associated with ecosystem disservices, such as diseases and property damage. Indeed, the effects of household‐scale characteristics on disease occurrence and transmission have long been recognized by epidemiologists and public health officials (Ishii et al. 1979; Gamble et al. 2023), both in terms of structural properties of houses and human occupant behaviours (e.g., having pets and hygiene habits; Dannemiller et al. 2016; Dom et al. 2026). Therefore, rather than relying solely on population‐ or landscape‐level averages and aggregated data, understanding the ecology of household biomes can provide unprecedented data to inform multilevel analyses in public health research (Diez‐Roux 2000), fostering more efficient and realistic diagnosis and interventions.

This historical focus on ecosystem disservices associated with household biodiversity has led to enduring efforts to eliminate indoor biodiversity (Martin et al. 2015; Dunn 2018), with nontrivial eco‐evolutionary and public health implications (Hartmann et al. 2016). However, it is impossible to keep houses lifeless, and most of this indoor biodiversity is harmless. Actually, many of these species are native (some are rarely found outdoors) (Bertone et al. 2016) and can provide ecosystem services to human health (Hanski et al. 2012) and well‐being, such as controlling vectors (e.g., spiders preying upon mosquitoes) and removing organic matter from hard‐to‐reach places (e.g., generalist ants foraging or scavenging). Weighting the costs and benefits of household biodiversity for human lives—and how this balance varies across houses, socioeconomic realms, and biogeographic regions—remains underexplored. For instance, whether and when increased household biodiversity is linked to more ecosystem services than disservices is unknown. However, to manage and optimize household ecosystem services effectively, we first need to deepen our understanding of the natural history and interactions within our home ecosystem.

## 5 What's Next?

Understanding the structure and functioning of household ecosystems is an interdisciplinary endeavour that requires integrating biodiversity sciences with economics, social sciences, architecture, and public health. Integrating indoor biodiversity into modern urban ecology (Pickett et al. 2016; MacGregor‐Fors et al. 2026) is critical to understanding the ecological and evolutionary consequences of human settlements on biodiversity, particularly how our social patterns (Alberti et al. 2020; Schell et al. 2020) reverberate on organisms that live and coevolved with us. Naturally, there is an evident potential to benefit from citizen science approaches (Mason et al. 2025; Allf et al. 2026). Complementarily, technology now enables the collection of unprecedented information about elusive species and cryptic interactions (Tosa et al. 2021). Early on, ecologists eschewed urban environments because they are interdisciplinary and their functioning is inherently tied to the complex social fabric of human societies. Now, we have the tools and teams necessary to make the cross‐disciplinary study of household ecosystems an opportunity rather than a challenge.

The word ecology derives from the Greek *oikos* (home) and *logos* (learning about), so we encourage ecologists to turn their attention to the biodiversity and interactions within *oikos*. Catalysing new ideas, creative methods, and modern tools for understanding the ecology of our homes can not only advance fundamental questions in urban ecology but also inform broader societal concerns and help connect people with the biodiversity we interact with most.

## Author Contributions

R.C.‐P. and R.R.D. conceived the idea. R.C.‐P. wrote the manuscript with input from R.R.D.

## Funding

RCP is supported by the Serrapilheira Institute (grant number Serra‐R‐2011‐37572) and grant no. 2020/11953‐2, São Paulo Research Foundation (FAPESP).

## Data Availability

This Viewpoint manuscript does not use data or code.
